# A safe combined nephrectomy and right lobectomy using the liver hanging maneuver for huge renal cell carcinoma directly invading the right lobe of the liver: report of a case

**DOI:** 10.1007/s00595-013-0693-3

**Published:** 2013-09-19

**Authors:** Masanori Yoshimatsu, Ken Shirabe, Yoshihiro Nagao, Noboru Harada, Hideaki Uchiyama, Tomoharu Yoshizumi, Akinobu Taketomi, Tetsuo Ikeda, Katsunori Tatsugami, Yoshihiko Maehara

**Affiliations:** 1 Department of Surgery and Science, Graduate School of Medical Sciences, Kyushu University, 3-1-1 Maidashi, Higashi-ku, Fukuoka, 812-8582 Japan; 2 Department of Urology, Graduate School of Medical Sciences, Kyushu University, Fukuoka, Japan

## Abstract

We herein discuss a patient who underwent simultaneous combined right nephrectomy and right lobectomy of the liver. A 64-year-old male was diagnosed with a huge right renal cell carcinoma (RCC), 13 cm in diameter, which was invading directly into the right hepatic lobe. This type of RCC has been rarely reported, and an anterior approach using the liver hanging maneuver was extremely useful during hepatic parenchymal dissection. The liver parenchymal dissection was performed prior to mobilization of the liver, because the mobilization of the right lobe of the liver was impossible. During the hepatic parenchymal resection, the liver was suspended with the tape and transected, and thereafter, retroperitoneal dissection, nephrectomy and right lobectomy of the liver were completed. The patient was discharged from the hospital on the 12th postoperative day with an uneventful clinical course. The anterior approach using the liver hanging maneuver during hepatic parenchymal resection can be safe and feasible for huge RCC invading the right hepatic lobe.

## Introduction

Simultaneous nephrectomy and major hepatectomy is an uncommon surgical technique. The indications for this operation in previous reports were adrenocortical carcinoma, a germ cell tumor, benign cysts, renal cell carcinoma (RCC) with liver metastasis and locally advanced RCC with direct extension into the adjacent liver parenchyma [1]. Among these indications, locally advanced RCC with direct extension into the right lobe of the liver was the most common indication [1].

During conventional right lobectomy of the liver, the right lobe of the liver is generally mobilized completely, with the right hepatic vein controlled outside the liver prior to parenchymal transection [2–4], and this conventional approach is helpful in reducing the amount of blood loss [5]. However, mobilization of the right hepatic lobe cannot be performed in some cases when the tumor is huge. The anterior approach is often adopted for patients requiring difficult major right hepatic resection for hepatocellular carcinoma [6].

We herein report the case of a patient who underwent combined nephrectomy and right hepatectomy by an anterior approach using Belghiti’s hanging maneuver for a huge RCC invading the right hepatic lobe.

## Case report

**Fig. 1:**
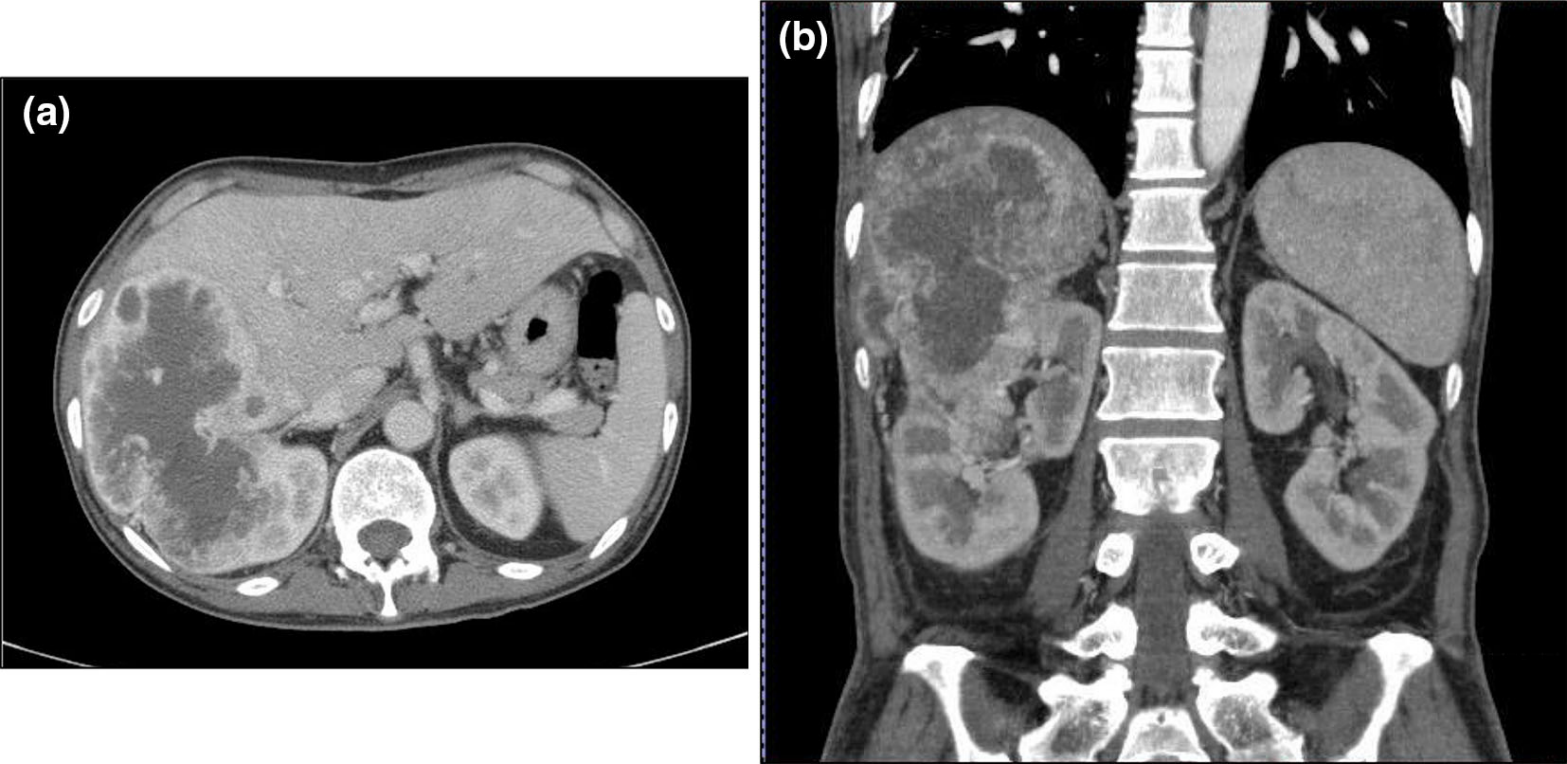
**a** A transverse and **b** a coronal computed tomography image. Preoperative computed tomography showed that there was a huge renal cell carcinoma (13 cm in diameter), invading the right lobe of the liver

We planned combined nephrectomy and right lobectomy of the liver for a 64-year-old male whose underlying disease was right RCC invading the right lobe of the liver. The tumor was 13 cm in diameter, and almost the whole right lobe was replaced by the invading tumor (Fig. 1).

The operation was performed after neoadjuvant chemotherapy using Sunitinib which was administered for advanced RCC. Exposure of the abdominal cavity was carried out through a reversed L incision from the skin to the peritoneum. First, the posterior peritoneum was opened, and the right kidney was mobilized from the retroperitoneal space. The right renal artery, vein and urethra were ligated and divided. Further dissection of the right adrenal gland and right kidney could not be done, because the right lobe of the liver interrupted the exposure of the upper side of the kidney and right adrenal gland.

Cholecystectomy was performed, and then the right hepatic artery and first branch of the right portal vein were ligated and dissected. The mobilization of the right lobe of the liver was impossible, so we performed the liver parenchymal transection prior to mobilization of the liver. The space between the right and middle hepatic veins was dissected, and then a long clamp was gently inserted in a cephalad direction at the 10–11 o’clock position, which was the dissected space between the right and middle hepatic veins.

**Fig. 2:**
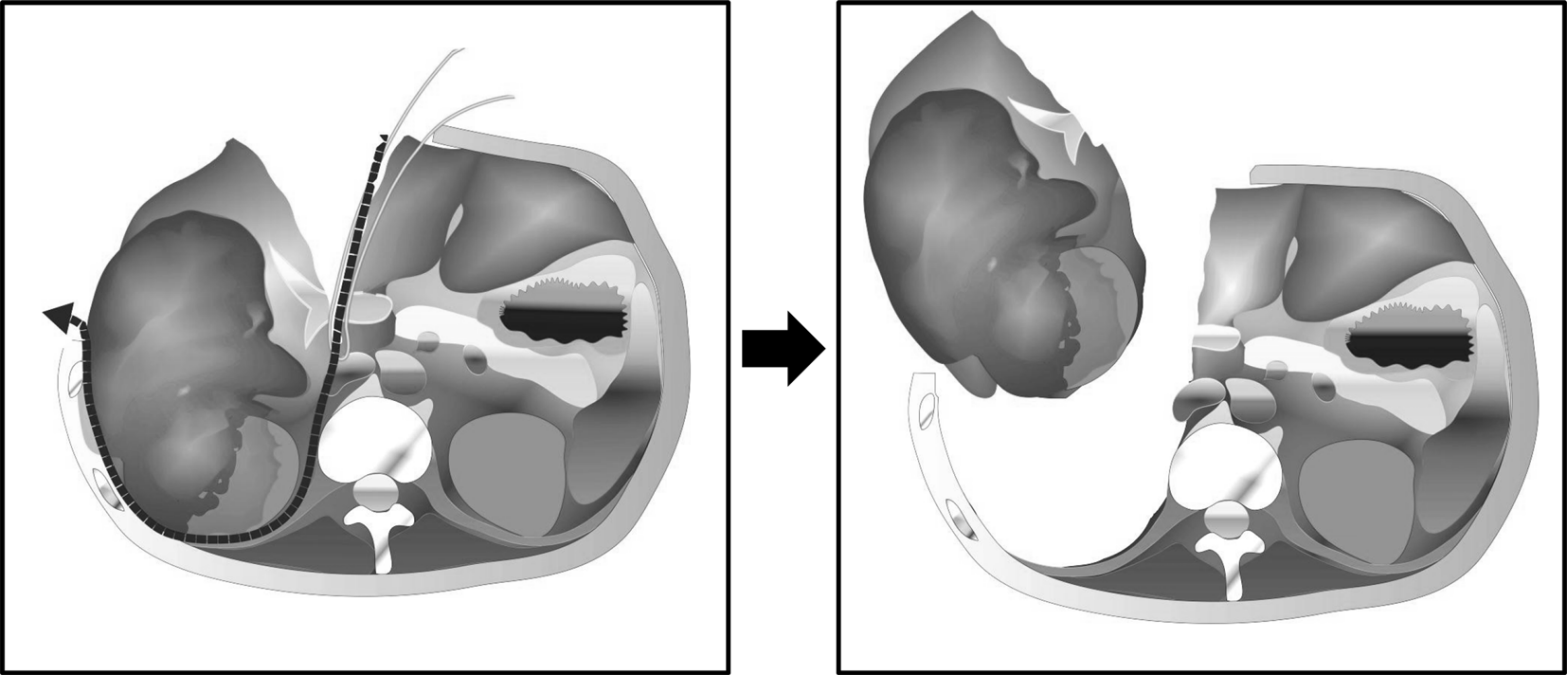
Scheme: The liver hanging maneuver and anterior approach during hepatic parenchymal dissection enabled a safe combined right nephrectomy and resection of the right lobe of the liver

**Fig. 3:**
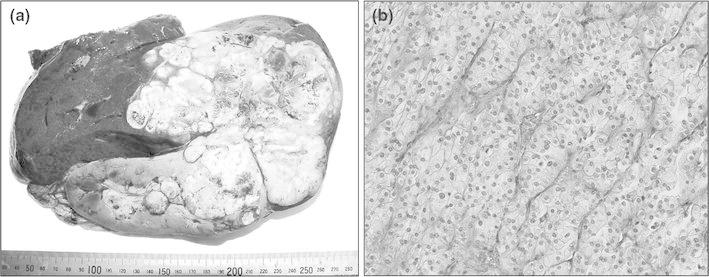
**a** The resected specimens of the right kidney and right lobe of the liver. **b** Histological specimens of the tumor showed a proliferation of carcinoma cells with clear to eosinophilic cytoplasm arranged in a sheet-like pattern, with nests and focally fascicular proliferation of spindle-shaped cells, together with massive necrosis, indicating renal clear cell carcinoma with sarcomatoid changes and direct invasion into the liver

When the tip of the clamp reached the space between the veins, two cotton tapes were clenched in the tips of the clamp. The clamp was pulled down through the entire length of the retrohepatic inferior vena cava, placing the tape in the retrohepatic space. The liver parenchyma was suspended on the tape. The liver parenchymal was then transected using a Cavitron Ultrasonic Surgical Aspirator (CUSA: Valleylab Inc., Boulder, CO, USA) along the demarcation line. Intermittent vascular inflow occlusion (Pringle Maneuver) was applied twice for 15-min intervals. Intraoperative ultrasonography was performed to assess the vascular and biliary anatomy, as well as the tumor distribution. Suspending the liver with the tape enabled us to control the bleeding at the time of the liver parenchymal dissection (Fig. 2). After completion of the parenchymal dissection, the short hepatic veins to the right lobe of the liver and right adrenal gland were ligated and divided. The right hepatic vein was divided with an Echelon 45 Endopath stapler (Ethicon Inc., NJ, USA) after liver parenchymal dissection. Retroperitoneal dissection of the upper kidney and resection of the right adrenal gland were done, and the combined nephrectomy and right hepatectomy were completed. The blood loss during surgery was 1,925 ml, and the resected weight of the right kidney and right hepatic lobe was 1,970 g (Fig. 3a). The histological specimens (Fig. 3b) indicated renal clear cell carcinoma with sarcomatoid changes and direct invasion to the liver. The patient’s postoperative course was uneventful, and he was discharged from the hospital on the 12th day after the operation.

## Discussion

In this report, we presented the case of a patient who underwent combined nephrectomy and right hepatectomy using the anterior approach, because the tumor was too large to be mobilized and rotated. Combined nephrectomy and major hepatectomy is an uncommon surgical technique. The anterior approach is a surgical procedure that can be employed without rotation and mobilization of the liver before parenchymal transection. Lai [7] employed the anterior approach for major hepatectomy when rotation and mobilization of the liver was difficult. Belghiti [8] developed an anterior approach using the liver hanging maneuver technique. Using this technique, the liver is suspended with tape during the procedure, which enables control of the bleeding in the deeper parenchymal plane and guides the direction of the anatomic parenchymal transection. A nasogastric tube, a soft silicon multitubular drain, or a cotton tape is usually used as the tape for the liver hanging maneuver [10], and Belghiti [9] originally recommended using a soft silicon multitubular drain. We used two cotton tapes for the liver hanging maneuver, because the material was relatively handy, hard to slip, and easy to fold when passed between the liver parenchyma and the IVC.

Recently, Yezhelyev [1] reviewed the cases of patients who underwent simultaneous nephrectomy with major hepatectomy, and demonstrated that this procedure can be performed with acceptable mobility and mortality. Among the 37 cases, 29 patients had RCC, and all of them had RCC in the right kidney. Among the 29 patients with RCC, 14 patients had direct invasion to the right lobe of the liver. In this type of RCC, the mobilization of the right lobe of the liver prior to parenchymal dissection is usually difficult, because *en bloc* resection of right kidney and right lobe of the liver is necessary, and the sizes of the right kidney and invaded right lobe of the liver are large. The anterior approach using the liver hanging maneuver is an indispensable technique in such cases. It has been shown that a tumor invading the inferior vena cava (IVC) was a contraindication for the liver hanging maneuver [9], and RCC rarely invades the IVC. Therefore, the liver hanging maneuver can be applied easily and is feasible. Furthermore, advanced RCC and adrenocortical carcinoma tend to be accompanied by tumor thrombus in the IVC. Yezhelyev [1] showed that in eight of 18 patients with RCC, adrenocortical and germ cell carcinoma, the tumor was accompanied by IVC thrombus. The anterior approach using the liver hanging maneuver would therefore be a good technique even in these patients.

Previously published reports described twenty patients who underwent combined nephrectomy and major hepatectomy for RCC directly invading into the right lobe of the liver, like the present case (Table 1). This is the third case of a combination of the anterior approach and hanging maneuver for huge RCC in the literature, because Yezhelyev [1] reported two cases. We wanted to examine the clinicopathological findings of RCC like this case, but there were no descriptions in the previous studies. In our institute, the anterior approach is indicated when rotation and mobilization of the right liver is considered to be difficult, and the liver hanging maneuver is indicated when right hepatectomy is performed for hepatocellular carcinoma in patients without contraindications, such as tumor infiltration into the retrohepatic avascular spaces or inferior vena cava and adhesions between the inferior vena cava and liver. Therefore, the anterior approach using the liver hanging maneuver was indicated in this case.

**Table 1:** Published reports of combined major hepatectomy and nephrectomy for right RCC directly extending into the right lobe of the liver

Author	Year	*N*	Sites of metastasis or extension	AA + LHM
Bennett [14]	1995	2	• Direct liver extension	–
Fujisaki [15]	1997	3	• Direct liver extension and liver metastasis	–
Kawata [16]	2000	2	• Direct liver extension, IVC thrombus and left adrenal metastasis	–
			• Direct liver extension and IVC thrombus	
Johnin [17]	2001	2	• Direct liver extension	–
Alves [18]	2003	2	• Direct liver extension and liver metastasis (S2,3,4,5,6,7,8)	–
			• Direct liver extension and liver metastasis (S2,3,7,8)	
Wong [19]	2006	1	• Direct invasion into the liver, diaphragm, lung and adrenal gland	–
Dorado [20]	2007	1	• Direct liver extension	–
Yezhelyev [1]	2008	7	• Direct liver extension	2
			• Two patients showed IVC thrombus	
Current	2010	1	• Direct liver extension	1

*AA* *+* *LHM* anterior approach using liver hanging maneuver

In the previously reported cases (Table 1), two patients underwent an anterior approach using the liver hanging maneuver to avoid tumor rupture. It might have been difficult to accomplish such operations without using this approach, so these patients were also indicated for this unusual procedure. In terms of the other cases, there were four cases with inferior vena cava tumor thrombus, but these reports did not describe any obvious tumor infiltration into the retrohepatic avascular spaces or inferior vena cava. If there were no adhesions between the inferior vena cava and the liver, the anterior approach with the liver hanging maneuver would be indicated for all cases to control bleeding at the deeper parenchymal plane and to guide the direction of the anatomical parenchymal transection.

In this case, the patient underwent neoadjuvant chemotherapy using Sunitinib before surgery. Some reports [11, 12] have indicated that there are surgical morbidities associated with using the tyrosine kinase inhibitor, Sunitinib, as neoadjuvant chemotherapy. Thomas [11] reported that surgical resection of RCC after targeted therapy is feasible with low morbidity in most patients, but that significant complications can occur, raising concerns about the possible compromise of tissue and/or vascular integrity, including a significant risk of hemorrhage under a concomitant liver resection and wound complications. Harshman [12] reported that there was no significant increase in perioperative complications, however, he showed a significant increase in the development of intraoperative adhesions. In the present case, there were no intraoperative or postoperative complications, such as severe hemorrhage, however, moderate adhesions were seen between the omentum and peritoneum. This might have been due to the effects of neoadjuvant chemotherapy. Powles [13] reported that 38 of 52 (73 %) patients obtained a clinical benefit from adjuvant therapy (by RECIST) before surgery. He reported a 6 % partial response rate, while none of the patients became ineligible due to local progression. In the case of locally advanced RCC invading the liver, neoadjuvant chemotherapy using Sunitinib, followed by combined nephrectomy and hepatectomy, could be a feasible and reasonable strategy.

In conclusion, a combination of nephrectomy and right hepatectomy by the anterior approach using Belghiti’s liver hanging maneuver appears to be safe and feasible for patients with huge renal cell carcinoma directly invading the right lobe of the liver.
